# Supplementary material for: Cerebellar nuclei excitatory neurons regulate developmental scaling of presynaptic Purkinje cell number and organ growth
Source: eLife. 2019 Nov 19;8:e50617. doi: 10.7554/eLife.50617 (PMC6890462; doi:10.7554/eLife.50617)
Supplement: Figure 1—source data 1. [file elife-50617-fig1-data1.pdf]

Source Data_1				
**Figure**	**Test performed**	**P-Value**	**Multiple comparisons**	
Fig. 1G	Two-way ANOVA	F (1, 6) = 43.14, P=0.0006	Vermis	0.7725
			Paravermis	0.0004
			Hemisphere	<0.0001
Fig. 1H	Two-way ANOVA	$F_{(1, 9)}$=398.277, P<0.0001	ASec	<0.0001
			CSec	<0.0001
			PSec	0.1498
Fig.1I	Two-way ANOVA	$F_{(1, 9)}$=0.2269, P=0.64	ASec	0.69
			CSec	0.9661
			PSec	0.9955
Fig. 1J	Two-way ANOVA	F (1, 9) = 0.2269, P=0.6452	ASec	0.6918
			CSec	0.9661
			PSec	0.9955
Fig. 1L	Two-way ANOVA	F (1, 15) = 72.52 P<0.0001	ASec	<0.0001
			CSec	<0.0001
			PSec	0.3064
Fig. 1M	Two-way ANOVA	F (1, 15) =0.2583 P=0.6187	ASec	0.9484
			CSec	0.8998
			PSec	0.8512
Fig.1N	Two-way ANOVA	F (1, 9) = 0.8772 P=0.3734	ASec	0.3167
			CSec	0.7412
			PSec	0.6820
Fig. 1O	Two-way ANOVA	F (1, 9) = 28.4 P=0.0005	ASec	0.0284
			CSec	0.0031
			PSec	0.5937
Fig. 1Q	Two-way ANOVA	F (1, 12) = 32.29 P=0.0001	MN	0.0058
			IN	0.0001
			LN	0.8003
Fig. 2A	Two-way ANOVA	F (1, 25) = 14.23 P=0.0009	D1 run1	>0.9999

			D1 run2	0.1858
			D1 run3	0.2107
			D2 run1	0.2708
			D2 run2	0.2367
			D2 run3	0.1525
			D3 run1	0.0036
			D3 run2	0.1440
			D3 run3	0.1104
Fig. 2B	Student's t-test	t(25)=3.772 P=0.0009	N/A	
Fig. 2C	Student's t-test	t(26)=0.9552 P=0.34	N/A	
Fig. 2D	Two-way ANOVA	F (1, 75) =8.227 P=0.0054	Stride	0.0023
			Sway	0.8102
			Stance	0.8834
Fig. 3C	Student's t-test	aMN: t(4)=4.492 P=0.0109<br>pMN: t(4)=4.412 P=0.0116	N/A	
Fig. 5C	Two-way ANOVA	F (1, 24) = 8.042 P=0.0091	eCN+GCP	0.0213
			eCN	0.0169
			GCP	0.8873
Fig. 5D	Two-way ANOVA	F (1, 6) = 12.88 P=0.0115	Vermis	0.0016
			Paravermis	0.0289
			Hemisphere	0.5858
Fig. 5E	Two-way ANOVA	F (1, 10) = 8.34 P=0.0162	Vermis	0.0082
			Paravermis	0.1806
			Hemisphere	0.8096
Fig. 5F	Two-way ANOVA	F (1, 18) = 22.07 P=0.0002	ASec	0.0005
			CSec	0.0848

			PSec	0.6617
Fig. 5G	Two-way ANOVA	F (1, 18) = 2.652 P=0.1208	ASec	0.4868
			CSec	0.5646
			PSec	0.9899
Fig. 5H	Two-way ANOVA	F (1, 6) = 0.1723 P=0.6925	ASec	0.9768
			CSec	0.9966
			PCec	0.9879
Fig. 5I	Two-way ANOVA	F (1, 18) = 32.51 P<0.0001	ASec	<0.0001
			CSec	0.0153
			PSec	0.8086
Fig. 5J	Two-way ANOVA	F (1, 18) =0.0185 P=0.8933	ASec	>0.9999
			CSec	0.5136
			PSec	0.6586
Fig. 5K	Two-way ANOVA	F (1, 18) =0.06465 P=0.8022	ASec	0.9932
			CSec	0.9818
			PSec	0.9346
Fig. 5L	Two-way ANOVA	F (1, 18) = 0.002591 P=0.9600	ASec	0.7930
			CSec	0.8317
			PSec	>0.9999
Fig. 5M	Two-way ANOVA	F (1, 36) = 12.4 P=0.0012	ASec	0.0056
			CSec	0.2819
			PSec	0.6417
Fig. 5N	Two-way ANOVA	F (1, 36) = 1.511 P=0.2269	ASec	0.7862
			CSec	0.9789
			PSec	0.7394

Fig. 5O	Two-way ANOVA	F (1, 36) = 0.135 P=0.7155	ASec	0.7730
			CSec	0.9979
			PSec	0.9715
Fig. 5P	Two-way ANOVA	F (1, 36) = 13.5 P=0.0008	ASec	0.0048
			CSec	0.0543
			PSec	0.9512
Fig. 5Q	Two-way ANOVA	F (1, 36) = 0.6296 P=0.4327	ASec	0.5705
			CSec	0.7100
			PSec	0.5812
Fig. 5R	Two-way ANOVA	F (1, 36) = 3.092 P=0.0872	ASec	0.9382
			CSec	0.5312
			PSec	0.5021
Fig. 5S	Two-way ANOVA	F (1, 36) = 0.5289 P=0.4718	ASec	0.7400
			CSec	>0.9999
			PSec	0.9805
Fig. 6B	Two-way ANOVA	F (1, 24) = 10.26 P=0.0038	eCN+GCP	0.0105
			eCN	0.0008
			GCP	0.5910
Fig. 6E	One sampe t-tests	t(3)=3.894	MN (eCN+GCP)	0.0300
		t(3)=4.919	IN (eCN+GCP)	0.0161
		t(3)=0.2765	LN (eCN+GCP)	0.8001
		t(6)=10.31, df=6	MN (eCN)	<0.0001
		t(6)=12.32, df=6	IN (eCN)	<0.0001
		t(6)=0.8325, df=6	LN (eCN)	0.4370

Fig. 6C	Two-way ANOVA	F (1, 120) = 63.33 P<0.0001		
Fig. 6D	Two-way ANOVA	F (1, 240) = 104.4 P<0.0001		
Fig. 6E	Student's t-test	t(6)=0.84 P=0.43	N/A	
Fig. 6F	Student's t-test	t(6)=2.88 P=0.02	N/A	
Fig. 7E	Student's t-test	t(4)=0.84, P=0.44	N/A	
Fig. 7F	Student's t-test	t(4)=3.08, P=0.036	N/A	
Fig. 7S	Two-way ANOVA	F (1, 42) = 2.182 P=0.1471		
Fig. 7T	Two-way ANOVA	F (1, 42) = 23.67 P<0.0001		
Fig. 7U	Student's t-test	t(4)=0.437, P=0.68	N/A	
Fig. 7V	Student's t-test	t(4)=8.96, P=0.0009	N/A	
Fig. 8B	Student's t-test	t(4)=7.6, P=0.0016	N/A	
Fig. 8C	Two-way ANOVA	F (1, 88) = 52.48 P<0.0001		
Fig. 8H	Two-way ANOVA	F (1, 4) = 19.47 P=0.0116	Vermis	0.0024
			Paravermis	0.0330
			Hemisphere	0.0339
Fig. 8I	Two-way ANOVA	F (1, 6) = 675.2 P<0.0001	Vermis	<0.0001
			Paravermis	<0.0001
			Hemisphere	<0.0001
Fig. 8J	Student's t-test	t(4)=3.165, P=0.034	N/A	
Fig. 8K	Two-way ANOVA	F (1, 12) = 0.1364 P=0.7184	Vermis	0.9847
			Paravermis	0.9046
			Hemisphere	0.3594
Fig. 8L	Two-way ANOVA	F (1, 12) = 2.578 P=0.1343	Vermis	0.4274
			Paravermis	0.8176
			Hemisphere	0.9453

Fig. 8M	Student's t-test	t(4)=1.408, P=0.23	N/A	
Fig. 8R	Student's t-test	(4)=0.922, P=0.4	N/A	
Figure 1_fig. supp1A	Two-way ANOVA	F (1, 9) = 44.48 P<0.0001	ASec	0.0134
			CSec	0.0002
			PSec	0.8443
Figure 1_fig. supp1B	Two-way ANOVA	$F_{(1,9)}$=98.8, P<0.0001	ASec	0.0001
			CSec	0.0002
			PSec	0.7396
Figure 1_fig. supp1C	Two-way ANOVA	$F_{(1,9)}$=278.3, P<0.0001	ASec	0.0001
			CSec	0.0001
			PSec	0.2706
Figure 1_fig. supp1D	Two-way ANOVA	F (1, 12) = 17.32 P=0.0013	ASec	0.0470
			CSec	0.0002
			PSec	0.3781
Figure 1_fig. supp1E	Two-way ANOVA	F (1, 12) = 41.63 P<0.0001	ASec	0.0202
			CSec	<0.0001
			PSec	0.8472
Figure 1_fig. supp1F	Two-way ANOVA	F (1, 12) = 42.43 P<0.0001	ASec	0.0008
			CSec	0.0009
			PSec	0.5895
Figure 1_fig. supp1G	Two-way ANOVA	F (1, 12) = 1.72 P=0.2142	ASec	0.9914
			CSec	0.9955
			PSec	0.2662
Figure 1_fig. supp1H	Two-way ANOVA	F (1, 12) =0.1698 P=0.6875	ASec	0.8758
			CSec	0.7713
			PSec	0.7597
Figure 1_fig. supp1I	Two-way ANOVA	F (1, 6) = 7.501 P=0.0338	ASec	0.1522
			CSec	0.4242

			PSec	0.8468
Figure 1_fig. supp1J	Two-way ANOVA	F (1, 6) =0.02607 P=0.8770	ASec	0.5575
			CSec	0.5659
			PSec	0.1667
Figure 1_fig. supp1K	Two-way ANOVA	F (1, 6) = 4.284 P=0.0839	ASec	0.9969
			CSec	0.5816
			PSec	0.1317
Figure 1_fig. supp1L	Two-way ANOVA	F (1, 6) = 2.241 P=0.1850	ASec	0.5676
			CSec	0.8843
			PSec	0.9190
Figure 1_fig. supp1M	Student's t-test	t(5)=6.397 P=0.014	N/A	
Figure 1_fig. supp2C	Two-way ANOVA	F (1, 12) = 0.01486 P=0.9050	ASec	0.8807
			CSec	0.9996
			PSec	0.8227
Figure 2_fig. sup1A	Two-way ANOVA	F (1, 225) = 42.34 P<0.0001	Day1 run1	0.9761
			Day1 run2	0.2416
			Day1 run3	0.0372
			Day2 run1	0.3866
			Day2 run2	0.1943
			Day2 run3	0.5126
			Day3 run1	0.0352
			Day3 run2	0.2269
			Day1 run3	0.1559
Figure 2_fig. sup1B	Student's t-test	t(15)=2.683 P=0.02	N/A	

Figure 2_fig. sup1C	Student’s t-test	t(15)=3.656 P=0.002	N/A	
Figure 2_fig. sup1E	Two-way ANOVA	F (1, 75) = 16.97 P<0.0001	Stride	<0.0001
			Sway	0.5801
			Stance	0.1657
Figure 5_fig. sup2A	Two-way ANOVA	F (1, 6) = 17.56 P=0.0057	ASec	0.0017
			CSec	0.2645
			PSec	0.5519
Figure 5_fig. sup2B	Two-way ANOVA	F (1, 18) = 5.284 P=0.0337	ASec	0.0324
			CSec	0.9999
			PSec	0.6498
Figure 5_fig. sup2C	Two-way ANOVA	F (1, 18) = 1.297 P=0.2697	ASec	0.9709
			CSec	0.9319
			PSec	0.6845
Figure 5_fig. sup2D	Two-way ANOVA	F (1, 18) = 0.008472 P=0.9277	ASec	0.5110
			CSec	0.2643
			PSec	0.9879
Figure 5_fig. sup2E	Two-way ANOVA	F (1, 18) = 0.1478 P=0.7051	ASec	0.5812
			CSec	0.6454
			PSec	0.8375
Figure 5_fig. sup2F	Two-way ANOVA	F (1, 18) = 2.328 P=0.1444	ASec	0.9949
			CSec	0.2276
			PSec	0.9185
Figure 5_fig. sup2G	Two-way ANOVA	F (1, 18) = 5.808 P=0.0269	ASec	0.1167
			CSec	0.6713
			PSec	0.7484

Figure 5_fig. sup2H	Two-way ANOVA	F (1, 18) = 5.633 P=0.0290	ASec	0.0103
			CSec	0.9998
			PSec	0.8085
Figure 5_fig. sup2I	Two-way ANOVA	F (1, 9) = 0.187 P=0.6756	ASec	0.9333
			CSec	0.9996
			PSec	0.9893
Figure 5_fig. sup2J	Two-way ANOVA	F (1, 30) = 0.7102 P=0.4061	ASec	0.9998
			CSec	0.9833
			PSec	0.6580
Figure 5_fig. sup2K	Two-way ANOVA	F (1, 30) = 1.469 P=0.2349	ASec	0.9999
			CSec	0.7447
			PSec	0.6153
Figure 5_fig. sup2L	Two-way ANOVA	F (1, 36) = 0.01968 P=0.8892	ASec	0.9997
			CSec	0.9998
			PSec	0.9934
Figure 5_fig. sup3A	Two-way ANOVA	F (1, 18) = 0.0002414 P=0.9878	ASec	0.9950
			CSec	>0.9999
			PSec	0.9884
Figure 5_fig. sup3B	Two-way ANOVA	F (1, 18) = 0.006082 P=0.9387	ASec	>0.9999
			CSec	>0.9999
			PSec	>0.9999
Figure 5_fig. sup3C	Two-way ANOVA	F (1, 18) = 1.211 P=0.2856	ASec	0.9897
			CSec	0.6696
			PSec	0.9207
			ASec	0.9994

Figure 5_fig. sup3D	Two-way ANOVA	F (1, 18) = 5.243 P=0.0343		
			CSec	0.0516
			PSec	0.4111
Figure 5_fig. sup3E	Two-way ANOVA	F (1, 18) = 30.22 P<0.0001	ASec	0.2963
			CSec	0.0002
			PSec	0.0363
Figure 5_fig. sup3F	Two-way ANOVA	F (1, 18) = 0.0002414 P=0.9878	ASec	0.9950
			CSec	>0.9999
			PSec	0.9884
Figure 5_fig. sup3G	Two-way ANOVA	F (1, 18) = 0.3863 P=0.5421	ASec	0.9341
			CSec	0.5013
			PSec	0.9866
Figure 5_fig. sup3H	Two-way ANOVA	F (1, 18) = 0.606 P=0.4464	ASec	0.9635
			CSec	0.7735
			PSec	>0.9999
Figure 5_fig. sup3I	Two-way ANOVA	F (1, 18) = 1.023 P=0.3252	ASec	0.9996
			CSec	0.5641
			PSec	0.8993
Figure 5_fig. sup3J	Two-way ANOVA	F (1, 18) = 1.542 P=0.2302	ASec	0.9924
			CSec	0.3247
			PSec	0.8277
Figure 5_fig. sup3K	Two-way ANOVA	F (1, 18) = 2.484 P=0.1325	ASec	0.9970
			CSec	0.2938
			PSec	0.5515
Figure 5_fig. sup3M	Student’s t-test	t(4)=8.283 P=0.0012	N/A	

Figure 6_fig. sup1C	Student's t-test	t(155)=12.64 P=0.0001	N/A
Figure7_fig. sup1A	Student's t-test	t(4)=0.2472 P=0.8169	N/A
Figure7_fig. sup1B	Student's t-test	t(4)=2.048 P=0.11	N/A
Figure7_fig. sup2D	Two-way ANOVA	F (1, 84) = 1.62 P=0.2067	
Figure7_fig. sup2E	Student's t-test	t(4)=1.248 P=0.2802	N/A
Figure7_fig. sup2I	Student's t-test	t(4)=0.3694 P=0.7306	N/A
Figure7_fig. sup2J	Student's t-test	t(4)=0.2448 P=0.8187	N/A
Figure7_fig. sup2N	Two-way ANOVA	F (1, 105) = 6.27 P=0.0138	
Figure7_fig. sup2O	Student's t-test	t(5)=1.757 P=0.1393	N/A
Figure7_fig. sup2T	Student's t-test	t(5)=0.08553 P=0.9352	N/A
Figure7_fig. sup2U	Student's t-test	t(5)=1.177 P=0.2923	N/A
